# EZH2 promotes progression of small cell lung cancer by suppressing the TGF-β-Smad-ASCL1 pathway

**DOI:** 10.1038/celldisc.2015.26

**Published:** 2015-09-22

**Authors:** Fumihiko Murai, Daizo Koinuma, Aya Shinozaki-Ushiku, Masashi Fukayama, Kohei Miyaozono, Shogo Ehata

**Affiliations:** 1 Department of Molecular Pathology, Graduate School of Medicine, The University of Tokyo, 7-3-1 Hongo, Bunkyo-ku, Tokyo, Japan; 2 Department of Pathology, Graduate School of Medicine, The University of Tokyo, 7-3-1 Hongo, Bunkyo-ku, Tokyo, Japan

**Keywords:** small cell lung cancer, epigenetics, apoptosis, EZH2, TGF-β, ASCL1, Smad

## Abstract

Transforming growth factor-β (TGF-β) induces apoptosis in many types of cancer cells and acts as a tumor suppressor. We performed a functional analysis of TGF-β signaling to identify a molecular mechanism that regulated survival in small cell lung cancer cells. Here, we found low expression of TGF-β type II receptor (TβRII) in most small cell lung cancer cells and tissues compared to normal lung epithelial cells and normal lung tissues, respectively. When wild-type TβRII was overexpressed in small cell lung cancer cells, TGF-β suppressed cell growth *in vitro* and tumor formation *in vivo* through induction of apoptosis. Components of polycomb repressive complex 2, including enhancer of zeste 2 (EZH2), were highly expressed in small cell lung cancer cells; this led to epigenetic silencing of TβRII expression and suppression of TGF-β-mediated apoptosis. Achaete-scute family bHLH transcription factor 1 (ASCL1; also known as ASH1), a Smad-dependent target of TGF-β, was found to induce survival in small cell lung cancer cells. Thus, EZH2 promoted small cell lung cancer progression by suppressing the TGF-β-Smad-ASCL1 pathway.

## Introduction

Lung cancer causes mortality more than any other type of cancer [1]. Lung cancer is mainly classified as either small cell lung cancer (SCLC) or non-small cell lung cancer (NSCLC), with incidences of ~15 and 84%, respectively [2]. SCLC, high-grade neuroendocrine tumors, has been reported to have the worst prognosis, with a 5-year survival rate of ~5% [3]. Those patients are mostly treated with anti-cancer drugs and/or radiation. However, a primary clinical issue is the acquisition of chemoresistance in SCLC cells [4]. Thus, it is essential to develop novel strategies for SCLC therapy. For successful drug discovery, it is important to find molecular mechanism(s) that maintain survival in SCLC cells.

Transforming growth factor (TGF)-β is a cytokine that exerts many biological functions. TGF-β binds to two different types of serine-threonine kinase receptors, termed type II (TβRII) and type I receptors (TβRI is also known as activin receptor-like kinase 5, ALK-5) expressed on the cell surface. Upon ligand binding, two TβRIIs and two TβRIs form a heterotetrameric complex, and this activated complex phosphorylates the receptor-regulated Smads (R-Smads), Smad2 and Smad3. The phosphorylated R-Smads form complexes with their common-partner Smad (Co-Smad), Smad4, and the R-Smad/Co-Smad complex translocates to the nucleus. Then, the complexes associate with various transcription factors and transcriptional co-activators or co-repressors, which in turn, regulate transcription of a wide spectrum of target genes [5–7].

TGF-β has been reported to have bi-directional roles in cancer progression [8]. TGF-β induces cell cycle arrest at G1 by regulating expression of cyclin-dependent kinase inhibitor 1 A (*CDKN1A*, also known as p21), *CDKN2B* (also known as p15), the v-myc avian myelocytomatosis viral oncogene homolog (*MYC*), and cell division cycle protein 25A (*CDC25A*) [9]. TGF-β also induces apoptosis in several types of cancer cells through multiple mechanisms [9, 10]. In ~80% of NSCLC tissues, expression of TβRII was lower than that of normal lung tissues [11]. It was also shown that restoration of TβRII into NSCLC cells inhibited their growth *in vitro* and *in vivo*. Conversely, TGF-β also plays critical roles in cancer metastasis via the epithelial-mesenchymal transition (EMT) [12]. Once EMT occurs in NSCLC cells, they acquire mesenchymal characteristics, which results in invasion and metastasis [13]. In NSCLC cells, thyroid transcription factor-1 (TTF-1) suppresses TGF-β-mediated EMT and inhibits cell migration and invasion [14]. Thus, the roles of TGF-β in the progression of NSCLC has been intensively studied. In contrast to NSCLC, the roles of TGF-β in the progression of SCLC have not been fully investigated. A few studies have reported that expression of *TGFBR2* (the gene that encodes TβRII) was decreased in some SCLC cells, but the mechanisms were not detailed [15, 16]. Therefore, the present study aimed to clarify the roles of TGF-β in SCLC cells, to identify the mechanisms involved in the downregulation of TβRII, and to identify novel TGF-β target genes in this type of cancer.

## Results

### Downregulation of TβRII expression in SCLC cells

**Figure 1:**
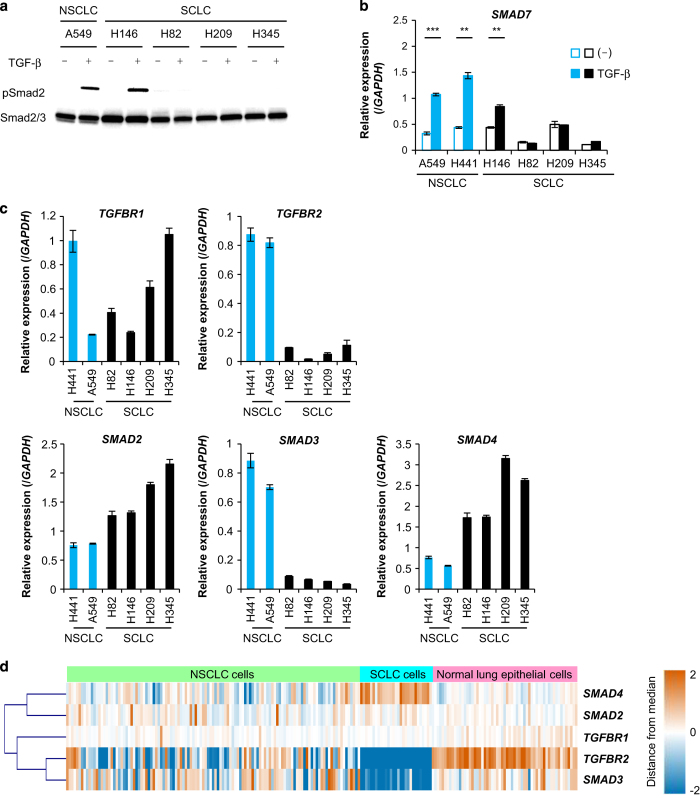
TGF-β signal transduction is attenuated in several SCLC cells due to decreased expression of TβRII. (**a** and **b**) SCLC and NSCLC cells were stimulated with TGF-β for 2 h. (**a**) Immunoblot of cell lysates probed with the indicated antibodies; (**b**) qRT-PCR analysis of *SMAD7* expression. Data represent mean±s.d. **P<0.01; ***P<0.001. (**c**) qRT-PCR analysis shows expression of TGF-β signaling components in SCLC and NSCLC cells. Data represent mean±s.d. (**d**) Comprehensive gene expression analysis from the NCBI GEO database (GSE32036) shows expression profiles of TGF-β signaling components in normal lung epithelial cells (*n*=59), SCLC cells (*n*=29) and NSCLC cells (*n*=119). Raw data were normalized by quantile algorithm. The color indicates the distance from the median of each row. GEO, gene expression omnibus; NCBI, National Center for Biotechnology Information; NSCLC, non-small cell lung cancer; SCLC, small cell lung cancer; TGF-β, transforming growth factor-β; qRT-PCR, quantitative real-time reverse transcription-PCR.

First, we investigated whether TGF-β signals were transduced in SCLC cells. Phosphorylation of Smad2 and induction of *SMAD7*, one of the direct targets of TGF-β, were examined in human SCLC cells (H146, H82, H209 and H345) and in NSCLC cells (A549 and H441) with immunoblotting and quantitative real-time reverse transcription-PCR (qRT-PCR) analyses. TGF-β-mediated phosphorylation of Smad2 was observed in H146 cells as well as in A549 and H441 cells (Figure 1a and data not shown, see Isogaya *et al *[17]). Induction of *SMAD7* by TGF-β was also observed in H146, A549 and H441 cells (Figure 1b). However, in the other SCLC cells, these responses were not induced by TGF-β. A qRT-PCR analysis also showed that expression of *TGFBR2* and *SMAD3* was decreased in SCLC cells, but other TGF-β signaling components, including *SMAD2*, *SMAD4* and *TGFBR1* (the gene that encodes TβRI), were expressed at normal levels in these cells (Figure 1c). These expression profiles were confirmed with comprehensive gene expression analysis data from the gene expression omnibus (GEO) of the National Center for Biotechnology Information (NCBI) with statistically significant differences (Figure 1d, and Supplementary Figure S1). Since TGF-β signal is transduced even in the low expression levels of Smad3 if Smad2 is expressed in H146 cells (Figure 1b), we assumed that TGF-β signal transduction was attenuated in SCLC cells through the decreased expression of TβRII, and therefore, we decided to focus on the roles of TβRII in SCLC in the present study.

### TβRII suppresses SCLC tumor growth through TGF-β-induced apoptosis

**Figure 2:**
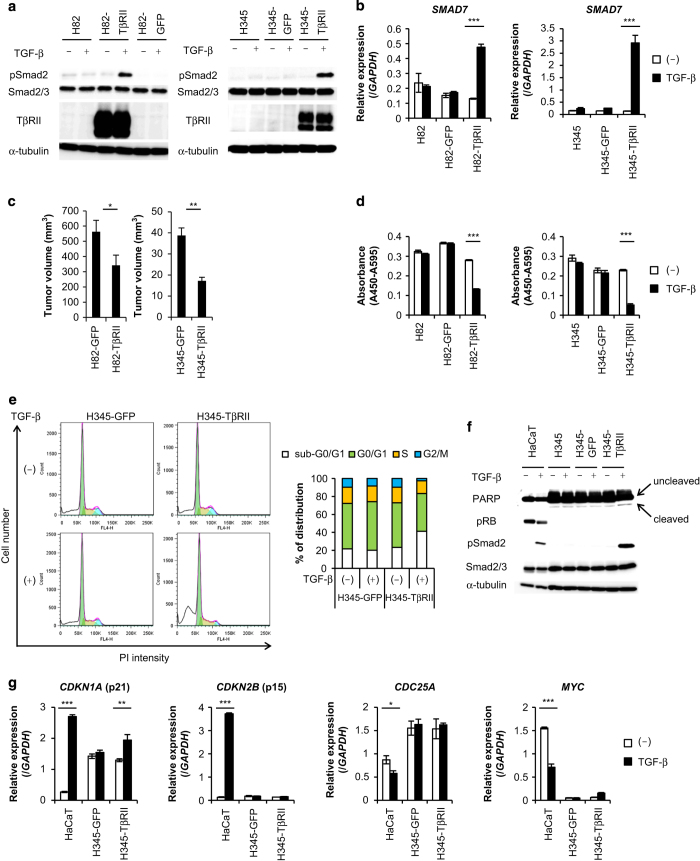
TβRII suppresses SCLC tumor growth through TGF-β-induced apoptosis. (**a** and **b**) SCLC cells were infected with lentivirus vectors encoding GFP (H82-GFP and H345-GFP) or TβRII (H82-TβRII and H345-TβRII). Cells were stimulated with TGF-β for 2 h. (**a**) Immunoblots of cell lysates probed with the indicated antibodies. (**b**) qRT-PCR analysis of *SMAD7* expression. Data represent mean±s.d. ***P<0.001. (**c**) Mice received subcutaneous transplantations of H82-GFP (*n*=15) and H82-TβRII cells (*n*=12) or H345-GFP (*n*=7) and H345-TβRII cells (*n*=9), and tumor volumes were measured 16 days (H82) or 17 days (H345) after transplantation. Data represent mean±s.e.m. *P<0.05; **P<0.01. (**d**) Cell proliferation assay. SCLC cells were stimulated with TGF-β for 6 days (H82) or 12 days (H345). Data represent mean±s.d. ***P<0.001. (**e**) Cell cycle analysis. (left panels) H345-GFP and H345-TβRII cells were unstimulated (top panels) or stimulated (bottom panels) with TGF-β for 12 days; the number of cells in each cell cycle stage is shown (color coding shown in right panel). (right panel) Percentage of cells in each cell cycle stage. (**f**) Immunoblots of cell lysates probed with the indicated antibodies. SCLC and control HaCaT cells were stimulated with TGF-β for 48 h (HaCaT) or 12 days (H345). (**g**) qRT-PCR analysis shows cell cycle-related gene expression. H345-GFP, H345-TβRII and HaCaT cells were stimulated with TGF-β for 2 h (*MYC*) or 24 h (*CDKN1A*, *CDKN2B* and *CDC25A*). Data represent mean±s.d. *P<0.05; **P<0.01; ***P<0.001. GFP, green fluorescent protein; SCLC, small cell lung cancer; TGF-β, transforming growth factor-β; qRT-PCR, quantitative real-time reverse transcription-PCR.

To examine the roles of TGF-β in SCLC progression, wild-type TβRII was introduced into H82 cells (H82-TβRII cells) or H345 cells (H345-TβRII cells) with lentiviral vectors. Both phosphorylation of Smad2 and induction of *SMAD7* by TGF-β were observed in TβRII-expressing cancer cells, but not in control SCLC cells that expressed green fluorescent protein (GFP) alone (H82-GFP cells and H345-GFP cells; Figure 2a and b). Thus, TGF-β signal transduction was successfully recovered by expressing TβRII. These cells were subcutaneously xenografted into nude mice to examine tumor growth *in vivo*. Tumor formation was decreased in mice injected with H82-TβRII cells and H345-TβRII cells, compared with mice xenografted with the control cells (Figure 2c). Although the expression of *TGFBR2* mRNA was low (Figure 1c) and the TβRII protein was not detected by immunoblot analysis (data not shown), Smad-dependent TGF-β signal was transduced in H146 cells (Figures 1a and b), suggesting that a low level of TβRII protein may be functioning in these cells. Thus, a GFP-tagged dominant-negative form of TβRII (dnTβRII) was overexpressed in H146 cells (H146-dnTβRII cells; Supplementary Figure S2a). Both phosphorylation of Smad2 and induction of *SMAD7* were inhibited by the introduction of dnTβRII (Supplementary Figures S2b and S2c). When these cells were subcutaneously xenografted into mice, tumor formation was accelerated in mice injected with H146-dnTβRII cells compared with those injected with H146-GFP cells (Supplementary Figure S2d). These results suggested that TGF-β may act as a tumor suppressor *in vivo*. We assessed angiogenesis in these tumor tissues by staining for CD31 expression (also known as platelet/endothelial cell adhesion molecule 1, PECAM1). However, there was no remarkable difference in CD31 expression between H146-GFP and H146-dnTβRII cells xenografted tissues (Supplementary Figure S2e). This finding suggested that tumor suppression was mediated by the effect of TGF-β on SCLC cells, not by its effect on angiogenesis in the tumor microenvironment.

We postulated that TGF-β might suppress the proliferative activity of SCLC cells. When we restored TβRII expression in these cells, TGF-β significantly suppressed the *in vitro* proliferation of H82 cells and H345 cells (Figure 2d). Moreover, dnTβRII expression canceled TGF-β-mediated growth inhibition in H146 cells (Supplementary Figure S2f). Cell cycle analysis revealed that TGF-β increased the sub-G0/G1 population in H345-TβRII cells compared with H345-GFP cells (Figure 2e). TGF-β also induced the cleavage of poly (ADP-ribose) polymerase (PARP) in H345-TβRII cells (Figure 2f), which suggested that TGF-β decreased the number of SCLC cells by inducing apoptosis. TGF-β is also known to suppress proliferation of many types of cells by regulating CDK activators or inhibitors. We found that expression levels of *CDKN1A*, *CDKN2B, MYC* or *CDC25A* in H345-TβRII cells were not markedly altered by TGF-β (Figure 2g). However, in human keratinocyte HaCaT cells, TGF-β upregulated the expression of *CDKN1A* and *CDKN2B* and downregulated the expression of *MYC* and *CDC25A*. Moreover, expression of retinoblastoma protein (pRB) was not detected in H345 cells (Figure 2f). These results suggested that TGF-β suppressed proliferation of SCLC cells by inducing apoptosis, but not by regulating the cell cycle.

### Importance of EZH2-mediated silencing of TβRII for SCLC tumor formation

**Figure 3:**
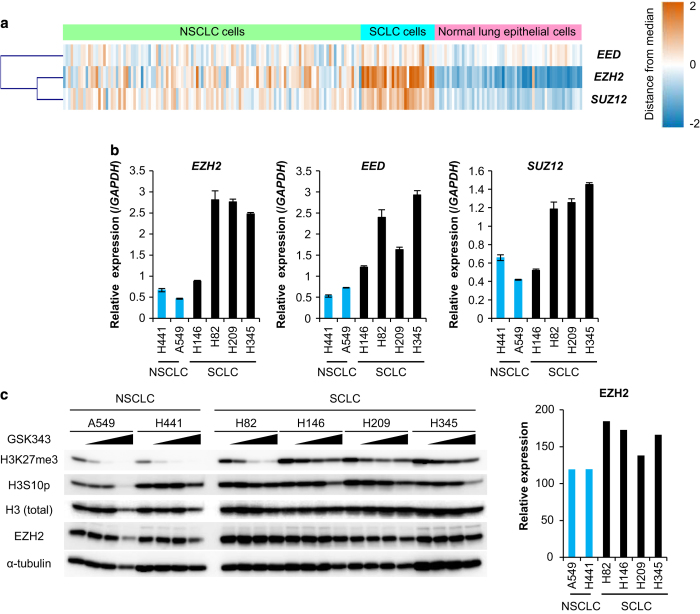
EZH2 is highly expressed in SCLC cells. (**a**) Expression profiles of PRC2 components in SCLC cells, based on the data in Figure 1d. The color indicates the distance from the median of each row. (**b**) qRT-PCR analysis shows expression of PRC2 components in SCLC and NSCLC cells. Data represent mean±s.d. (**c**) Immunoblot of cell lysates probed with the indicated antibodies. (left panels) SCLC and NSCLC cells were treated with GSK343 in a wide concentration range (0, 0.4, 2 and 10 μm) for 3 days. (right panel) Relative expression of EZH2 protein in each cell without GSK343 was quantified. NSCLC, non-small cell lung cancer; SCLC, small cell lung cancer; qRT-PCR, quantitative real-time reverse transcription-PCR.

Histone methyltransferase mediates methylation on lysine or arginine residues of histones of the H3 and H4 families to regulate transcription of various genes [18]. We postulated that, in SCLCs, the expression of TβRII might be epigenetically silenced through histone modification by histone methyltransferases. Therefore, we investigated histone methyltransferase expression in SCLC cells. Comprehensive gene expression analyses from the NCBI GEO data set revealed that SCLC cells and SCLC tissues displayed higher expression of the enhancer of zeste 2 (*EZH2*), SUZ12 polycomb repressive complex 2 subunit (*SUZ12*), and embryonic ectoderm development (*EED*) than normal lung epithelial cells and normal lung tissues, respectively (Figure 3a, and Supplementary Figure S3). We also found that their expressions were increased in SCLC cells but not in NSCLC cells (Figure 3b and c). These molecules associate to form the polycomb repressive complex 2 (PRC2), which inhibits gene transcription through methylation of lysine 27 in histone H3 (H3K27me3). High expression of H3K27me3 was observed in SCLC cells, which was attenuated by the treatment with an EZH2 inhibitor, GSK343 (Figure 3c), suggesting that PRC2 is implicated in transcriptional regulation of several genes in SCLC cells. H146 cells showed different expression profiles of the PRC2 complex from those in the other SCLC cells. The expression level of EZH2 protein was similar to those in the other SCLC cells, while the expression levels of EZH2, EED and SUZ12 mRNAs were lower than those in the other SCLC cells (Figures 3b and c).

**Figure 4:**
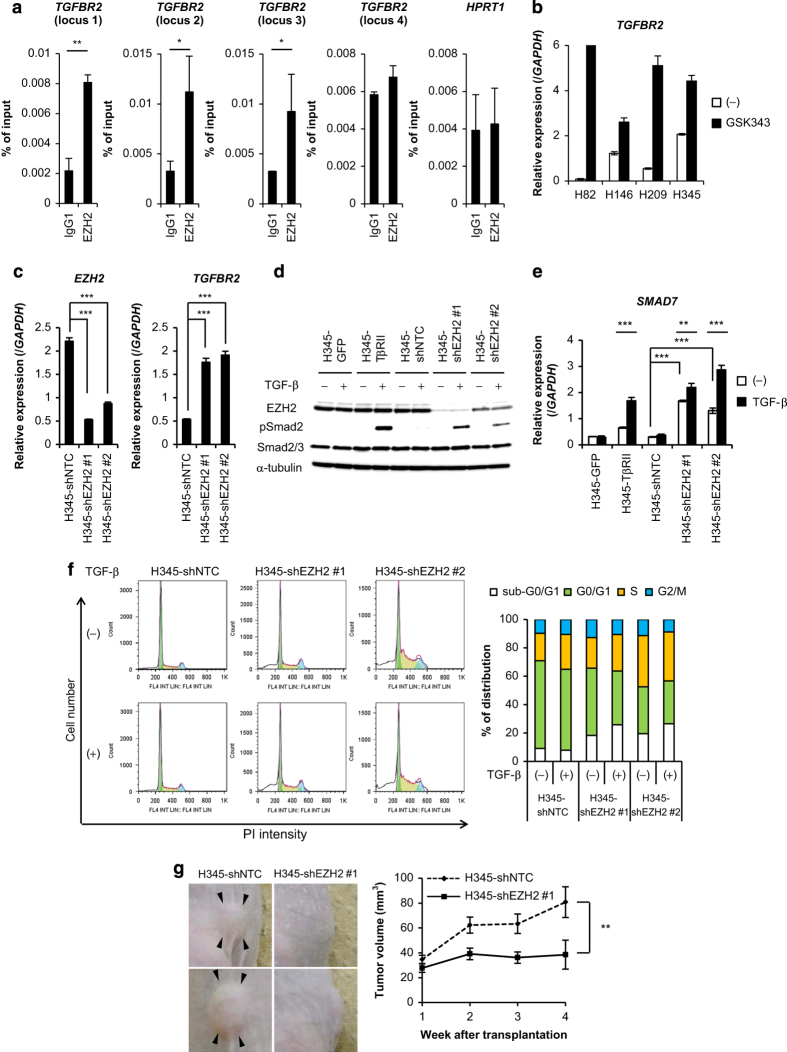
EZH2-mediated silencing of TβRII is required for SCLC tumor formation. (**a**) qRT-PCR analysis post-immunoprecipitation with anti-EZH2 antibody shows EZH2 enrichment in the *TGFBR2* locus of H345 cells. Hypoxanthine guanine phosphoribosyl transferase1 (*HPRT1*) was used as the negative control. Data represent mean±s.d. *TGFBR2* locus 1, chromosome 3: 30606416–30606492 bp; *TGFBR2* locus 2, chromosome 3: 30605676–30605771 bp; *TGFBR2* locus 3, chromosome 3: 30604616–30604714 bp; *TGFBR2* locus 4, chromosome 3: 30603967–30604061 bp. *P<0.05; **P<0.01. (**b**) qRT-PCR analysis shows *TGFBR2* expression. SCLC cells were treated with GSK343 (10 μM) for 7 days. Data represent mean±s.d. (**c**) qRT-PCR analysis shows *EZH2* and *TGFBR2* expression. H345 cells were infected with lentivirus vectors with control shRNA (H345-shNTC) or shRNA that targeted EZH2 (H345-shEZH2). Data represent mean±s.d. ***P<0.001. (**d**) Immunoblot of cell lysates from cells in (c) stimulated with TGF-β for 2 h and probed with the indicated antibodies. H345-GFP cells and H345-TβRII cells were negative and positive controls, respectively. (**e**) qRT-PCR analysis shows *SMAD7* expression. Cells in (c) were stimulated with TGF-β for 4 h. Data represent mean±s.d. H345-GFP cells and H345-TβRII cells were negative and positive controls, respectively. **P<0.01; ***P<0.001. (**f**) Cell cycle analysis of cells in (c) stimulated with TGF-β for 12 days. (left panels) The number of cells in each cell cycle stage is shown (color coding shown in right panel). (right panel) Percentage of cells in each cell cycle stage. (**g**) Mice received subcutaneous transplants of H345-shNTC cells (*n*=7) or H345-shEZH2 #1 cells (*n*=7). (left panels) Representative photographs 4 weeks after injection. Arrow heads indicate tumors. (right panel) Tumor volumes at the indicated time points. Data represent mean±s.e.m. **P<0.01. SCLC, small cell lung cancer; qRT-PCR, quantitative real-time reverse transcription-PCR.

In order to directly examine whether EZH2 is involved in downregulation of *TGFBR2*, chromatin immunoprecipitation (ChIP)-qRT-PCR analysis using anti-EZH2 antibody was performed (Figure 4a). EZH2 bound to the several loci in *TGFBR2* in H345 cells. Moreover, transcription of *TGFBR2* mRNA was increased in GSK343-treated SCLC cells (Figure 4b). When EZH2 expression was silenced in H345 cells with a short hairpin RNA (shRNA) (H345-shEZH2), the knockdown of EZH2 led to an increase in *TGFBR2* expression (Figure 4c); in turn, TGF-β induced Smad2 phosphorylation and *SMAD7* expression (Figures 4d and e). These results suggested that EZH2 played a critical role in downregulating TβRII in SCLC cells.

We also investigated whether EZH2 was important for TGF-β-mediated apoptosis and tumor formation in H345 cells. Cell cycle analysis showed that TGF-β increased the sub-G0/G1 population in H345-shEZH2 cells, but not in H345-shNTC cells (Figure 4f). Moreover, the ability of H345-shEZH2 cells to form tumors was attenuated, compared to that of H345-shNTC cells (Figure 4g).

### ASCL1 is negatively regulated by TGF-β in a Smad-dependent manner

**Figure 5:**
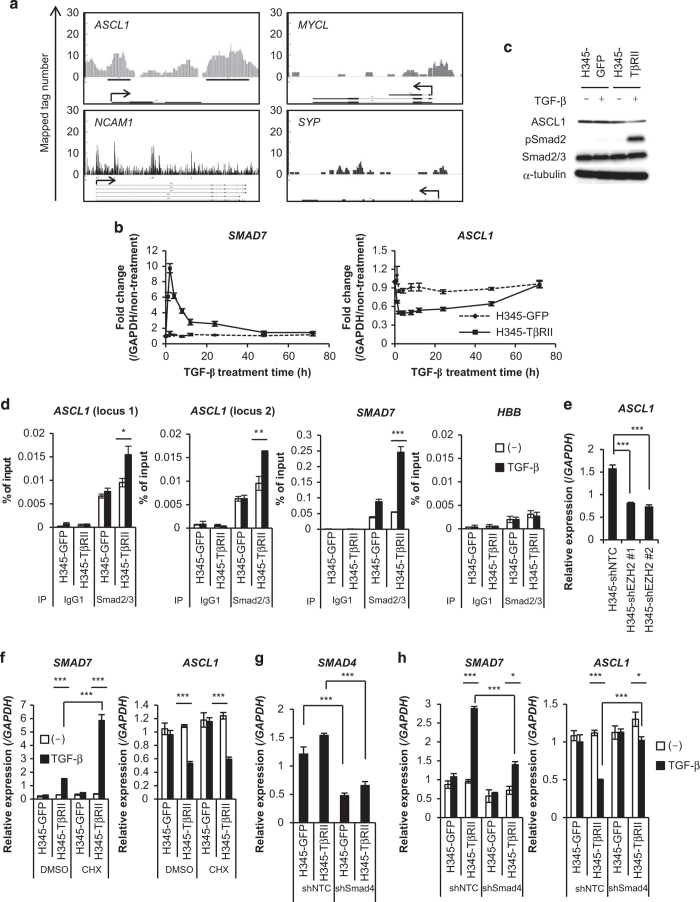
ASCL1 is negatively regulated by TGF-β in a Smad-dependent manner. (**a**) ChIP-seq analysis using anti-Smad2/3 antibody. H345-TβRII cells were stimulated with TGF-β for 1.5 h. Arrows indicate transcription start sites and direction. (**b**) qRT-PCR analysis shows *SMAD7* and *ASCL1* expression in H345-GFP and H345-TβRII cells after TGF-β stimulation for the indicated times. Data represent mean±s.d. (**c**) Immunoblot of cell lysates probed with the indicated antibodies. H345-GFP and H345-TβRII cells were stimulated with TGF-β for 12 days. (**d**) qRT-PCR analysis post-immunoprecipitation with anti-Smad2/3 antibody shows Smad2/3 enrichment. H345-GFP and H345-TβRII cells were stimulated with TGF-β for 1.5 h. Hemoglobin beta (*HBB*) was used as a negative control. Data represent mean±s.d. *ASCL1* locus 1, chromosome 12: 103351326–103352062 bp; *ASCL1* locus 2, chromosome 12: 103351326–103352062 bp. *P<0.05; **P<0.01. (**e**) qRT-PCR analysis shows *ASCL1* expression in indicated cells. Data represent mean±s.d. ***P<0.001. (**f**) qRT-PCR analysis shows *SMAD7* and *ASCL1* expression. H345-GFP and H345-TβRII cells were stimulated with TGF-β for 4 h after pre-treatment with CHX (3 μM) for 24 h. Data represent mean±s.d. ***P<0.001. (**g**) qRT-PCR analysis shows *SMAD4* expression. H345-GFP and H345-TβRII cells were infected with lentivirus vector with control shRNA (shNTC) or shRNA that targeted Smad4 (shSmad4). Data represent mean±s.d. (**h**) qRT-PCR analysis shows *SMAD7* and *ASCL1* expression. Cells in (g) were stimulated with TGF-β for 4 h. Data represent mean±s.d. *P<0.05; ***P<0.001. ChIP, chromatin immunoprecipitation; TGF-β, transforming growth factor-β; qRT-PCR, quantitative real-time reverse transcription-PCR.

We next attempted to identify TGF-β target genes that were involved in SCLC cell apoptosis. ChIP-sequencing (ChIP-seq) analysis was performed with an anti-Smad2/3 antibody in H345-TβRII cells to identify comprehensively Smad2/3-regulated genes. The characteristics of SCLC cells are thought to depend on the expression of several neuroendocrine-related genes, including the achaete-scute family basic helix-loop-helix transcription factor 1 (*ASCL1*, also known as *ASH1*), synaptophysin (*SYP*), neural cell adhesion molecule (*NCAM*) and v-myc avian myelocytomatosis viral oncogene lung carcinoma derived homolog (*MYCL*) [19]. Therefore, we focused on Smad2/3 binding to these gene loci. ChIP-seq analysis showed that, in the presence of TGF-β, Smad2/3 significantly bound to two loci of the *ASCL1* gene and several loci of the *NCAM1* gene, but not to the *SYP* locus or *MYCL* locus, in H345-TβRII cells (Figure 5a). Among the binding regions in the *NCAM1* locus, Smad2/3 strongly bound to the first intron. Comprehensive gene expression analysis from the NCBI GEO data sets showed that the mRNA levels of each of these neuroendocrine-related genes were elevated in SCLC cells and SCLC tissues, except for *SYP* in the SCLC tissue (Supplementary Figures S4a and S4b). Next, TGF-β regulation of these genes was assessed by qRT-PCR analysis (Figure 5b and Supplementary Figures S5a and S5b). In H345-TβRII cells, treatment with TGF-β caused *ASCL1* expression to decrease within 1 h, and it reached a minimum at 4 h. TGF-β also decreased *ASCL1* expression within 2 h in H146 cells. However, *MYCL*, *NCAM1* and *SYP* were not regulated by TGF-β in these cells. Moreover, TGF-β suppressed ASCL1 protein expression in SCLC cells (Figure 5c). In accordance with a previous analysis (Supplementary Figure S4a), in NSCLC cells, *ASCL1* was only weakly expressed, and it was not regulated by TGF-β (A549 and H441) (Supplementary Figure S5c). These results suggested that *ASCL1* was a TGF-β target, and regulation of *ASCL1* by TGF-β was specific for SCLC cells.

We then focused on the molecular mechanism underlying TGF-β-mediated transcriptional regulation of *ASCL1* in SCLC cells. ChIP-qRT-PCR analysis was performed to confirm whether Smad2/3 bound in the *ASCL1* locus. We found that TGF-β-stimulated H345-TβRII cells showed a sixfold-enrichment of Smad2/3 binding to these loci, compared with those to hemoglobin beta (*HBB*) locus (Figure 5d). In addition to the recovery of TβRII expression and TGF-β signal transduction in H345-shEZH2 cells (see Figures 4d and e), *ASCL1* expression was decreased in these cells (Figure 5e). Next, cycloheximide (CHX), a *de novo* protein synthesis inhibitor, was used to investigate whether *ASCL1* expression was directly or indirectly regulated by TGF-β. Even in the presence of CHX, TGF-β could upregulate *SMAD7* and downregulate *ASCL1* expression (Figure 5f). This result suggested that, similar to *SMAD7,* the expression of *ASCL1* was directly regulated by TGF-β.

TGF-β can activate both Smad and non-Smad pathways. To determine which pathway played a predominant role in regulating *ASCL1* expression, we used a shRNA (shSmad4) to silence the expression of Smad4 in H345-TβRII cells. The knockdown of Smad4 attenuated the induction of *SMAD7* by TGF-β (Figures 5g and h). Moreover, TGF-β-mediated downregulation of *ASCL1* expression was canceled in H345-TβRII cells by shSmad4, but not in cells infected with negative control shRNA (shNTC). These results suggested that TGF-β directly suppressed *ASCL1* expression in a Smad-dependent manner.

### ASCL1 promotes survival of SCLC cells

**Figure 6:**
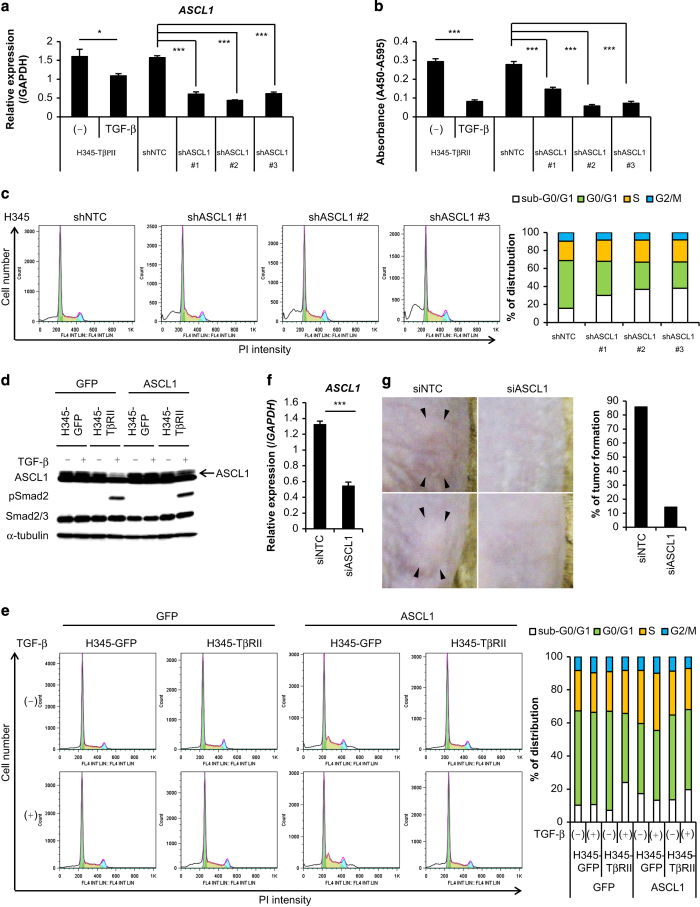
Downregulation of ASCL1 is important for TGF-β-mediated apoptosis in SCLC cells. (**a**) qRT-PCR analysis shows *ASCL1* expression. H345 cells were infected with lentivirus vectors encoding control shRNA (shNTC) or shRNA that targeted ASCL1 (shASCL1). H345-TβRII cells stimulated with TGF-β for 48 h served as control. Data represent mean±s.d. *P<0.05; ***P<0.001. (**b**) Cell proliferation assay. Cells in (a) were incubated for 12 days. Data represent mean±s.d. ***P<0.001. (**c**) Cell cycle analysis of cells in (b). (left panels) The number of cells in each cell cycle stage is shown (color coding shown in right panel). (right panel) Percentage of cells in each cell cycle stage. (**d**) Immunoblot of cell lysates probed with the indicated antibodies. H345-GFP and H345-TβRII cells were infected with lentivirus vectors encoding GFP alone or ASCL1, and then stimulated with TGF-β for 12 days. (**e**) Cell cycle analysis of cells in (d). (left panels) The number of cells in each cell cycle stage is shown (color coding shown in right panel). (right panel) Percentage of each cell cycle stage in indicated cells is shown. (**f**) qRT-PCR analysis shows *ASCL1* expression in H345 cells transfected with control siRNA (siNTC) or siRNA that targeted ASCL1 (siASCL1). *ASCL1* expression was determined 72 h post-transfection. Data represent mean±s.d. ***P<0.001. (**g**) Mice received subcutaneous transplants of cells in (f) (siNTC, *n*=7, siASCL1, *n*=7). (Left panels) Representative photographs; (right panel) incidence of tumor formation 2 weeks after injection. Arrow heads indicate tumors. GFP, green fluorescent protein; SCLC, small cell lung cancer; TGF-β, transforming growth factor-β; qRT-PCR, quantitative real-time reverse transcription-PCR.

Next, we determined whether negative regulation of *ASCL1* transcription was important for TGF-β-mediated apoptosis of SCLC cells. When expression of ASCL1 was silenced in H345 cells with shRNA (shASCL1) (Figure 6a), cell growth was inhibited by the induction of apoptosis (Figures 6b and c). Conversely, when ASCL1 was exogenously introduced by transferring lentiviral vectors into H345-TβRII cells, cell cycle analysis revealed that the TGF-β-mediated increase in the sub-G0/G1 population was attenuated (Figures 6d and e). These results suggested that ASCL1 had an important role in enabling SCLC cells to escape from TGF-β-induced apoptosis.

Next, we performed subcutaneous transplantations in mice to determine whether knocking down ASCL1 expression would suppress tumor growth or formation. We knocked down *ASCL1* expression by treating cells with small interfering RNA (siRNA) that targeted ASCL1 (siASCL1). Control cells were treated with negative control siRNA (siNTC) (Figure 6f). The siNTC treated-H345 cells formed tumors in six out of seven mice, but siASCL1-treated H345 cells formed tumors in only one out of seven mice (Figure 6g). These results suggested that ASCL1 was involved in SCLC tumor formation.

### EZH2-mediated silencing of TβRII in SCLC tissues

**Figure 7:**
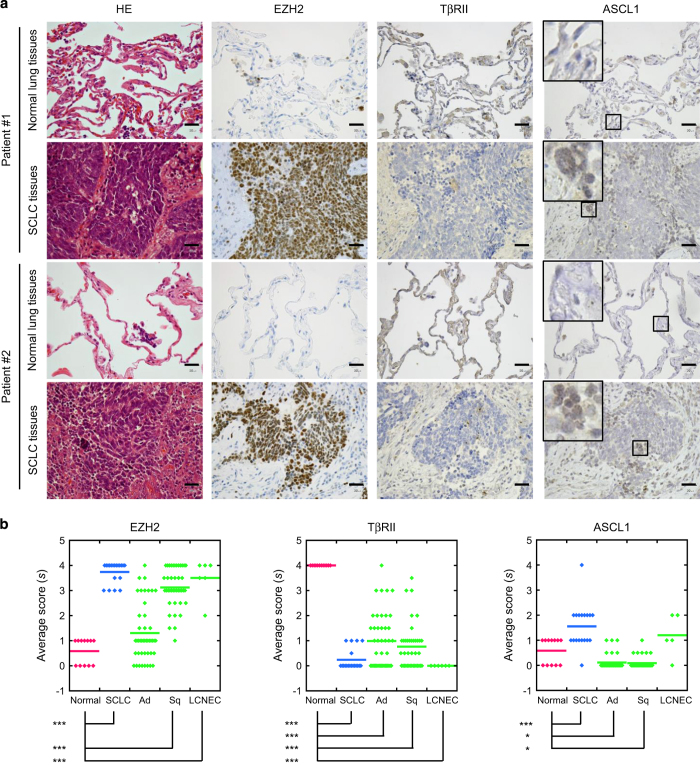
Expression profiles of EZH2, TβRII and ASCL1 in human normal lung tissues and human lung cancer tissues. (**a**) Lung tissues were stained with HE, anti-EZH2 antibody, anti-TβRII antibody and anti-ASCL1 antibody. Representative images show normal tissues (top and third rows) and SCLC tissues (second and bottom rows) from two patients, as indicated. (Insets) ASCL1 staining in boxed region is shown at high magnification. Scale bars are 30 μm. (**b**) Expression profiles of samples in (a) and Supplementary Figure S6 were analyzed by defining scores (*s*) that corresponded to the frequency of positive cells (*f*) in each sample, as follows: *s*=4 for 80⩽*f*⩽100; *s*=3 for 50⩽*f*<80; *s*=2 for 20⩽*f*<50; *s*=1 for 0<*f*<20; *s*=0 for *f*=0. Data represent means. *P<0.05; ***P<0.001. HE, hematoxylin-eosin; LCNEC, large cell neuroendocrine carcinoma; normal, normal lung; SCLC, small cell carcinoma; Ad, adenocarcinoma; Sq, squamous cell carcinoma.

Finally, we compared the expression of EZH2, TβRII and ASCL1 in SCLC tissues and NSCLC tissues to that in normal lung tissues (Supplementary Table S1). Immunohistochemical analysis showed that EZH2 was strongly expressed in the nuclei of all SCLC cells (Figure 7a). However, its expression was not observed in those of normal lung epithelial cells. In contrast, TβRII was weakly expressed in SCLC cells, while it was expressed on the surface of normal lung epithelial cells (Figure 7a). Although ASCL1 was also detected in nuclei of some SCLC cells, it was not observed in most of normal lung epithelial cells and other lung cancer cells (Figure 7a and Supplementary Figure S6). The expression profiles of EZH2, TβRII and ASCL1 are shown in Figure 7b. The number of positive samples, grouped according to expression frequency, is shown in Supplementary Table S2. When all lung tissues in Figure 7 were considered, there was a negative correlation between EZH2 and TβRII and a positive correlation between EZH2 and ASCL1 (Supplementary Table S3). These results supported the notion that EZH2 had a role in epigenetic silencing of TβRII in human SCLC tissues, and that the loss of TβRII upregulated the expression of ASCL1.

## Discussion

**Figure 8:**
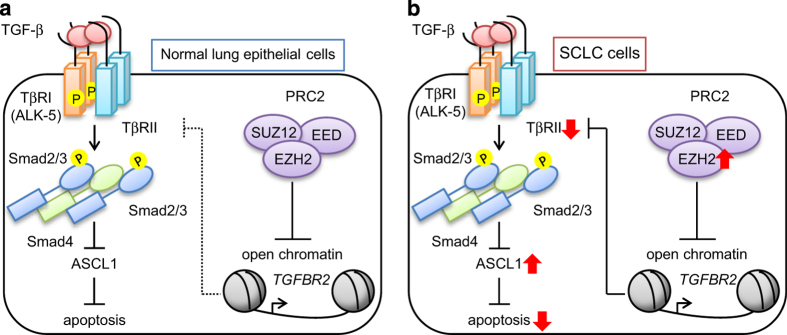
Disruption of TGF-β-mediated tumor suppression in SCLC cells. (**a**) In normal lung epithelial cells, TGF-β induces apoptosis through the suppression of ASCL1 expression in a Smad-dependent manner. (**b**) In SCLC cells, high EZH2 expression attenuates TβRII expression through histone H3K27 tri-methylation. Disruption of TGF-β signaling elevates ASCL1 expression, which in turn protects SCLC cells from apoptosis. SCLC, small cell lung cancer; TGF-β, transforming growth factor-β.

The present study clarified the tumor suppressive role of TGF-β in SCLCs. We showed that TβRII was expressed in normal lung epithelial cells, and that it inhibited abnormal cell growth by downregulating ASCL1 in a Smad-dependent manner (Figures 8a and b). However, EZH2 was highly expressed in SCLC cells, which epigenetically attenuated the expression of TβRII; thus, TGF-β signaling was suppressed, which resulted in high ASCL1 levels and progression of SCLC. These results suggested new therapeutic strategies for targeting EZH2 and ASCL1 in SCLC therapy.

We demonstrated that TGF-β inhibited proliferation of SCLC cells *in vivo* and *in vitro*. The SCLC cells used in this study carried mutations in the DNA binding region of *TP53*, and they did not express pro-apoptotic pRB, except for the H209 cells (Figure 2f, data not shown) [20]. Thus, TGF-β-mediated apoptosis in SCLC cells may occur independently of p53 and pRB. TGF-β causes cell cycle arrest at the G1 phase or apoptosis in many types of cancer cells [9]. For cell cycle arrest at the G1 phase, TGF-β regulates p21, p15, c-Myc and CDC25A in various types of cells, but it did not regulate these genes in SCLC cells. TGF-β was also reported to induce apoptosis by inducing expression of BCL2-like 11 (*BCL2L11*, also known as Bim), growth arrest and DNA-damage-inducible beta (*GADD45B*) and inositol polyphosphate-5-phosphatase 145 kDa (*INPP5D*, also known as SHIP) [9]. In contrast, our comprehensive gene expression analysis and ChIP-seq analysis showed that the TGF-β-mediated apoptosis in SCLC cells appeared to be independent of those genes (data not shown). Based on these observations, we attempted to identify novel target(s) for TGF-β, which could regulate survival in SCLC cells.

In many types of cancers, TβRII is dysfunctional through either genetic mutation or transcriptional repression [21–23]. The *TGFBR2* locus was shown to be mutated in the 10-adenine (A10) tract of exon 3 and serine-threonine kinase domain in some cancers [21–24]. Previous studies demonstrated that genetic mutations in the *TGFBR2* A10 tract or expression of a truncated TβRII were not common in SCLC [15, 25]. Moreover, loss of heterozygosity in chromosome 3, including in the *TGFBR2* locus, was not observed in SCLC [25]. Those studies indicated that genetic mutation was not a common mechanism for the dysfunction of TβRII in SCLC. In contrast, transcriptional repression of *TGFBR2* was reported in retinoblastoma and hematopoietic malignancies [23, 26].

In this study, we showed that PRC2 components were highly expressed in most of the SCLC cells, and that increased EZH2 expression caused silencing of TβRII expression in SCLC cells. Sato *et al *[27]. reported that many kinds of genes, such as *JUB*, *PTRF*, *DMKN*, *AXL* and *EPHB4*, were identified as targets for EZH2 in SCLC cells by ChIP-seq analysis. They also found that introduction of JUB inhibited cellular growth, suggesting that suppression of these genes by EZH2 other than *TGFBR2* might be involved in the growth of SCLC. Genetic alternations in *TP53* and *RB1* are commonly observed in patients with SCLC; consequently, these were considered as early events that triggered SCLC development [28–30]. EZH2 expression was upregulated in *Rb1* knockout MEF cells [31]. Based on those studies, our finding that EZH2 was highly expressed in SCLC suggests that EZH2 is an oncogenic factor. EZH2 was also reported to be highly expressed in breast cancer and prostate cancer [32, 33]; moreover, EZH2 inhibitors have been considered a promising therapy for certain types of tumors [34, 35]. A specific EZH2 inhibitor induced *TGFBR2* expression (Figure 4b); therefore, EZH2 inhibitors may also effectively eradicate SCLC cells by restoring TβRII expression, and thus, enabling TGF-β-mediated apoptosis.

TGF-β-target genes have been comprehensively identified in many kinds of tumors, including liver cancer, pancreatic cancer, NSCLC and breast cancer [17, 36–38], but rarely in neuroendocrine tumors. Identification of novel target(s) may improve our understanding of SCLC cell characteristics. In the present study, our comprehensive gene expression analysis and ChIP-seq analysis demonstrated that the TGF-β-induced apoptosis in SCLC cells could be attributed to negative regulation of ASCL1 by TGF-β. It was previously shown that *ASCL1* expression was inhibited by Notch signaling in SCLC cells [39]. This report was the first to reveal the mechanism underlying TGF-β regulation of ASCL1 expression. ASCL1, a member of the basic helix-loop-helix family transcription factors, has a crucial role in the differentiation of neural stem cells into neuronal lineages [40]. ASCL1 is also expressed in neuroendocrine tumors, especially in cases with poor prognoses [41–48]. In addition, several genes were identified as targets for ASCL1 [49]. Among them, miRNA-375 (miR-375) was supposed to inactivate Yes-associated protein (YAP)1 in SCLC [50]. In H345-TβRII cells, TGF-β exhibited a downgulation of ASCL1, followed by upregulation of primary-miR 375 (pri-miR-375) (data not shown). Thus, our present findings suggested that the TGF-β-Smad-ASCL1 pathway is important in SCLC progression, and that it may also have an important role in other neuroendocrine tumors.

## Materials and Methods

### Cell culture and reagents

Human SCLC H82, H146, H209, H345 cells and human NSCLC A549 and H441 cells were purchased from American Type Culture Collection (ATCC, Manassas, VA, USA) and cultured as recommended. Human skin keratinocyte HaCaT cells were previously described [51]. TGF-β3 (R & D Systems, Minneapolis, MN, USA) was reconstituted in 4 mm HCl and 0.1% bovine serum albumin (BSA, Sigma-Aldrich, St Louis, MO, USA) and used at a concentration of 1 ng ml^−1^. GSK343 (Sigma-Aldrich) and CHX (Sigma-Aldrich) were reconstituted in dimethyl sulfoxide. See also Supplementary Information.

### Cell proliferation assay

H82 cells (1×10^4^ cells), H146 cells (3×10^4^ cells) and H345 cells (3×10^4^ cells) were seeded on 12-well plates, and then stimulated with TGF-β. Cell proliferation was evaluated with Cell Count Reagent SF (Nacalai Tesque, Kyoto, Japan). Absorbance at 450 nm was measured with a Model 680 Microplate Reader (Bio-Rad, Melville, NY, USA), followed by subtraction of absorbance at 595 nm.

### *In vivo* tumor growth assay

*In vivo* experiments were performed as previously described [52]. The protocols were approved by the Animal Ethics Committee of The University of Tokyo (approval number: 2186). BALB/c *nu*/*nu* mice (4 weeks, male) were purchased from Charles River Laboratories (Yokohama, Japan). Cells were resuspended in culture media supplemented with 50% BD Matrigel (BD Bioscience, San Jose, CA, USA), and then subcutaneously injected into mice (3×10^6^ cells in 100 μl per mouse). For xenograft transplantations, H345 cells were treated with siRNA against ASCL1 for 72 h *in vitro*, followed by subcutaneous transplantation of ASCL1-silenced cells into mice.

### Gene expression analysis

Total RNA was extracted with the RNeasy Mini Kit (Qiagen, Valencia, CA, USA). Complementary DNA was synthesized with the random hexamer protocol described in the PrimeScript II 1st strand complementary DNA Synthesis Kit (Takara, Otsu, Japan). For qRT-PCR analysis, gene expression was quantified with the StepOne Plus Real time-PCR System (Life Technologies, Tokyo, Japan) and the Fast SYBR Green Master Mix (Life Technologies). The expression level of each gene was normalized to that of glyceraldehyde-3-phosphate dehydrogenase (*GAPDH*). Primer sequences are shown in Supplementary Table S4.

### Immunoblotting

Immunoblotting was previously described [53, 54]. Cells were lysed in radio-immunoprecipitation assay buffer (50 mm Tris-HCl (pH 8.0), 150 mm NaCl, 1% Nonidet P-40, 0.1% SDS, and 0.5% sodium deoxycholate) with the Complete Protease Inhibitor Cocktail (Roche Diagnostics, Tokyo, Japan) and an EDTA-free phosphatase inhibitor cocktail (Nacalai Tesque). Protein concentrations were quantified with the BCA Protein Assay (ThermoFisher Scientific, Yokohama, Japan). Equal amounts of total protein were applied to SDS-polyaclylamide gel electrophoresis, and transferred to Fluoro Trans W membrane (Pall, East Hills, NY, USA). Chemiluminescence images were captured on ImageQuant LAS4000 (Fujifilm, Tokyo, Japan). Image J software (NIH) was used to quantify blot band intensities in Figure 3c. See also Supplementary Information.

### Lentiviral vector construction and lentivirus production

A lentiviral vector system (provided by Dr Hiroyuki Miyoshi, RIKEN) was used to induce specific gene introduction and knockdown. For gene introduction, we inserted complementary DNAs encoding the human wild-type *TGFBR2*, *TGFBR2* with a truncated intracellular domain and a carboxy-terminal GFP tag (dnTβRII), and human wild-type *ASCL1*, into the entry vector, pENTR201 [55]. Then, pENTR201 vectors were inserted into the lentiviral destination vector, pCSII-EF-RfA or pCSII-CMV-RfA, as previously described [56]. Vectors encoding *GFP* were also generated as controls.

Similarly, shRNAs designed to knockdown a specified gene were inserted into the entry vector pENTR4-H1. Then, pENTR4-H1 vectors that carried shRNAs specific for human *ASCL1* or *EZH2* were inserted into the lentiviral destination vector, pCS-RfA-EG. The shRNA target sequences for gene knockdowns were obtained from Dharmacon siDESIGN Center (GE Healthcare, Piscataway, NJ, USA; Supplementary Table S5). Lentiviral vectors were produced as described previously [53]. Culture supernatant was concentrated with Lenti-X Concentrator (Clontech, Palo Alto, CA, USA), then used for lentiviral vector infections.

### siRNA

An Accell-siRNA SMARTpool specific for human ASCL1 was purchased from Dharmacon (GE Healthcare), and reconstituted in 1×siRNA buffer (100 μm, GE Healthcare). The siRNA target sequences are shown in Supplementary Table S6. Cells were treated with siRNA at a final concentration of 1 μm.

### Cell cycle analysis

After washing with phosphate-buffered saline, cells were fixed with ice-cold 70% EtOH in phosphate-buffered saline and stored for more than 16 h at −20 °C. The fixed cells were resuspended in phosphate-buffered saline containing 0.25 mg ml^−1^ RNase A, and then incubated at 37 °C for 1 h. Cells were labeled with 50 μg ml^−1^ propidium iodide (PI, Life Technologies) for 30 min at 4 °C; then, cell cycle analysis was performed with a Gallios Flow Cytometer (Beckman Coulter, Miami, FL, USA). The distribution of each cell cycle stage was analyzed with the FlowJo (Tomy Digital Biology, Tokyo, Japan) Watson Pragmatic cell cycle analysis program.

### ChIP-qRT-PCR analysis and ChIP-seq analysis

ChIP was previously described [51, 57]. See also Supplementary Information. ChIP-qRT-PCR analyses were performed with the StepOne Plus Real time-PCR System and FastStart Universal SYBR Green Master (Rox) (Roche). Primer sequences for each gene locus are shown in Supplementary Table S4.

For ChIP-seq analysis, total amounts of double stranded DNA were quantified with Qubit dsDNA HS Assay Kits (Life Technologies). Libraries were prepared with IonXpress Plus Fragment Library Kit (Life Technologies). Libraries were quantified with Ion Library Quantification Kit (Life Technologies). Emulsion PCR and product purification were performed with Ion PGM Template OT2 400 Kit (Life Technologies). The amplified samples were sequenced with Ion PGM Sequencer (Life Technologies) with Ion PGM Hi-Q Sequencing Kit. The acquired read tags were mapped onto the NCBI hg19 human genome assembly. Analyses of ChIP-seq data were previously described [58, 59]. The significant Smad2/3 binding region was calculated using CisGenome version 2 using default parameters except for window size (400 bp), cut-off counts (⩾10 reads (*P*=0.023516)), and step size (25 bp). Raw ChIP-seq and peak call data are available at GEO (GSE63871).

### Immunohistochemistry

Immunohistochemistry was previously described [52]. Formalin-fixed, paraffin-embedded human clinical tissues and a tissue microarray were obtained from patients at the University of Tokyo Hospital with informed consent. The protocol was approved by the Research Ethics Committee at the University of Tokyo, Graduate School of Medicine (approval number: G2211-(8) and 2381-(4)). Immunohistochemistry was performed with the Ventana (Roche) or the VECTASTAIN Elite ABC Kit (Vector Laboratories, Burlingame, CA, USA) for hand-staining. In hand-staining, sections were deparaffinized in xylene, and autoclaved in 10 mm citrate buffer (pH 6.0) for 10 min at 121 °C for antigen retrieval. Endogenous peroxidase was inactivated in 3% H_2_O_2_ diluted with methanol for 20 min. The sections were immunostained with primary antibodies, then with secondary antibody, and subjected to the avidin/biotinylated peroxidase complex reaction. The immunodetection substrate was 3,3′-Diaminobenzidine (DAB, Vector Laboratories). Quantification was performed by counting the number of positive cells in each sample.

### Statistical analysis

Comparisons between samples were performed with the Student’s *t* test after the *F*-test. Comparisons between groups were performed with the analysis of variance; one-way analysis of variance (Tukey’s method) was applied to comprehensive gene expression analyses and immunohistochemical analyses. The repeated measure analysis of variance was applied to *in vivo* experiments. Significant differences were defined as *P*<0.05.
