# Supplementary figures and images for: EZH2 promotes progression of small cell lung cancer by suppressing the TGF-β-Smad-ASCL1 pathway
Source: Cell Discov. 2015 Sep 22;1:15026–. doi: 10.1038/celldisc.2015.26 (PMC4860843; doi:10.1038/celldisc.2015.26)

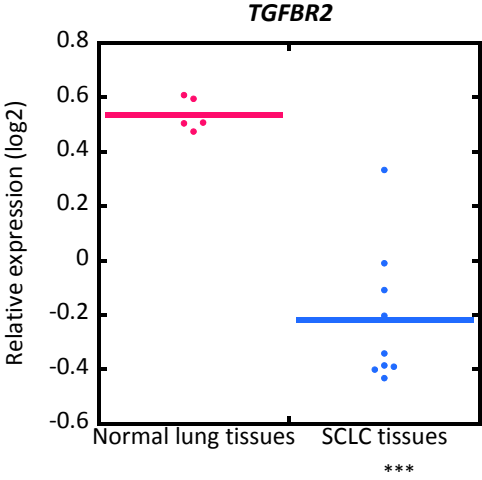

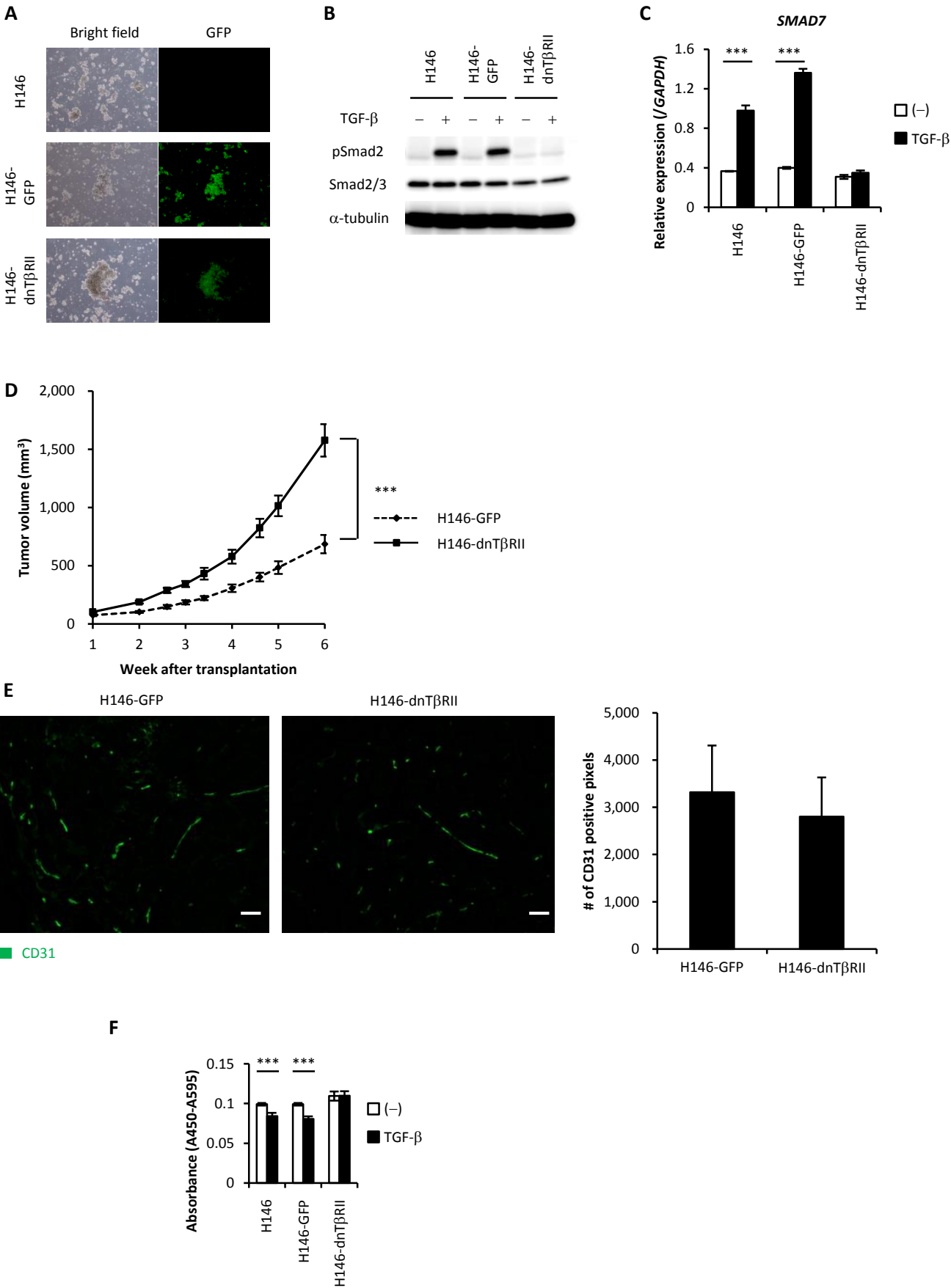

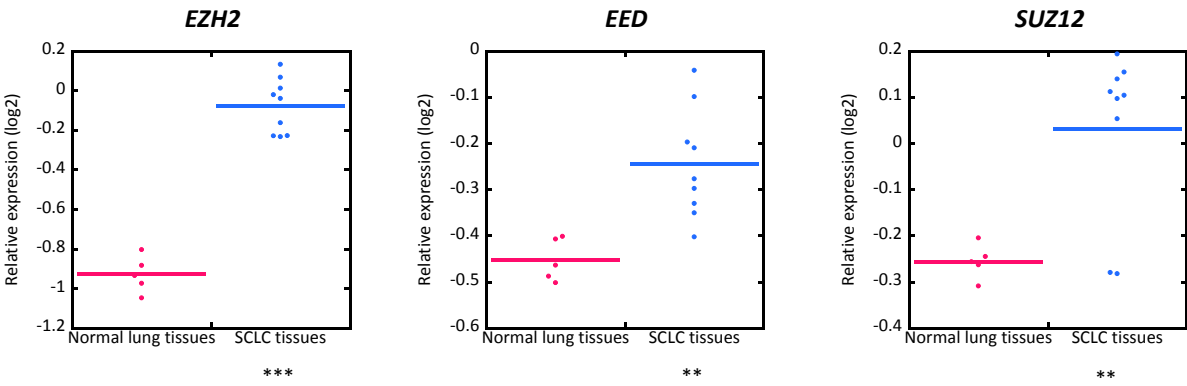

A

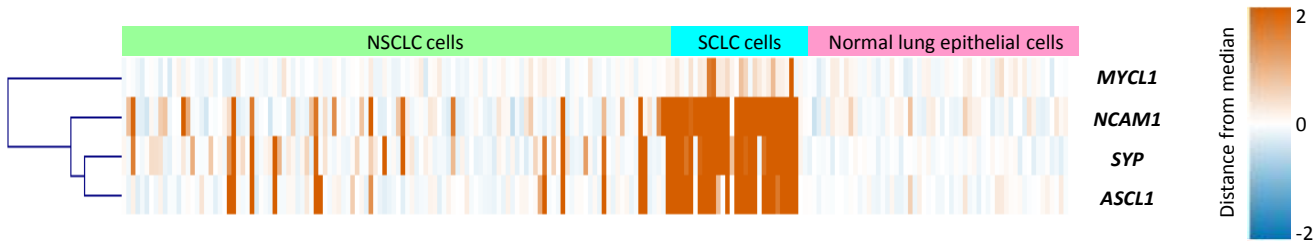

B

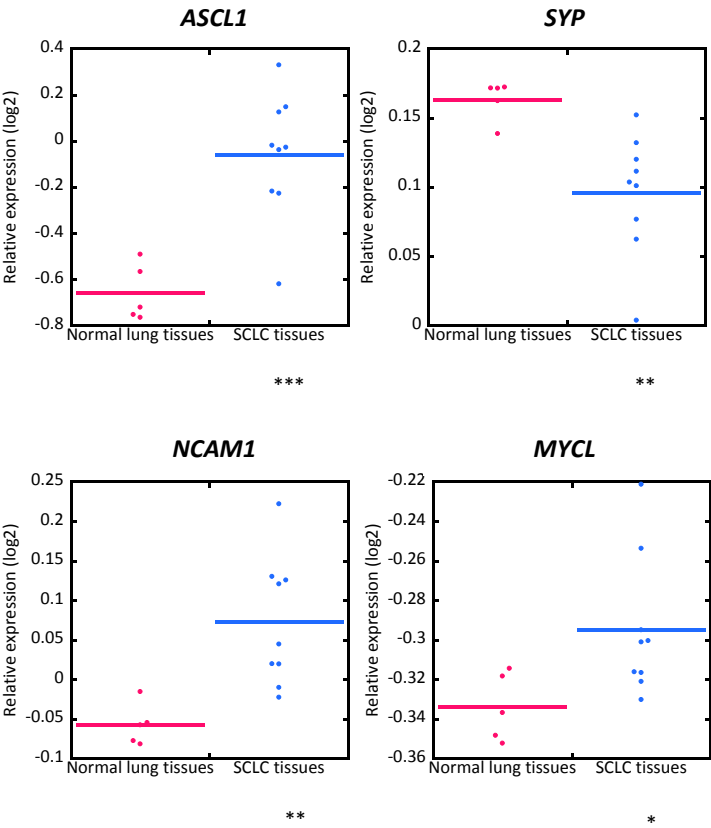

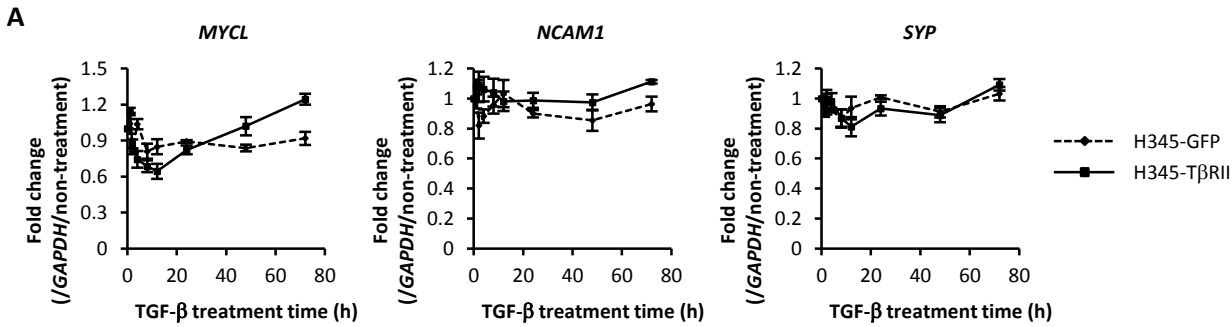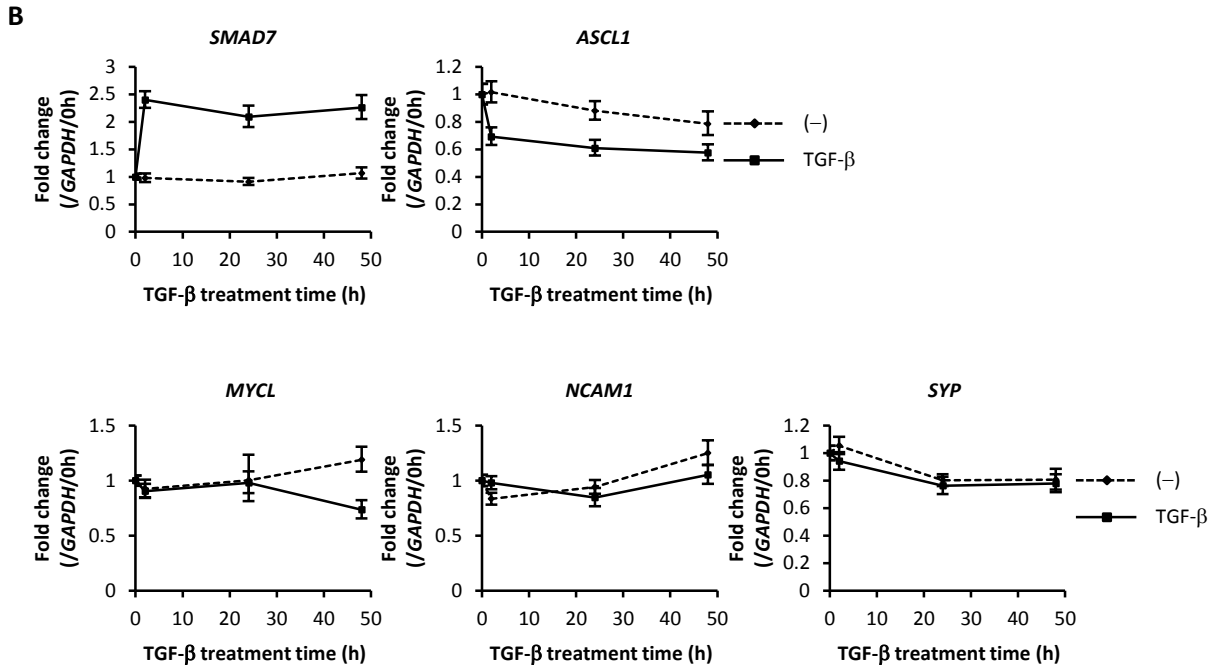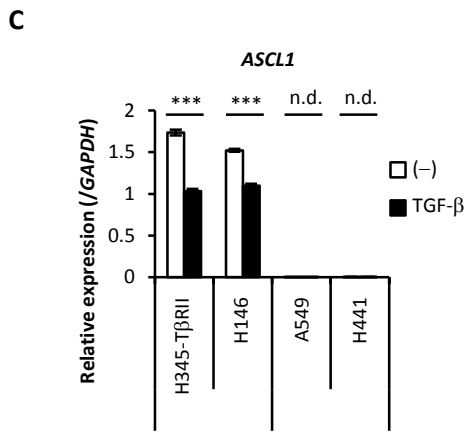

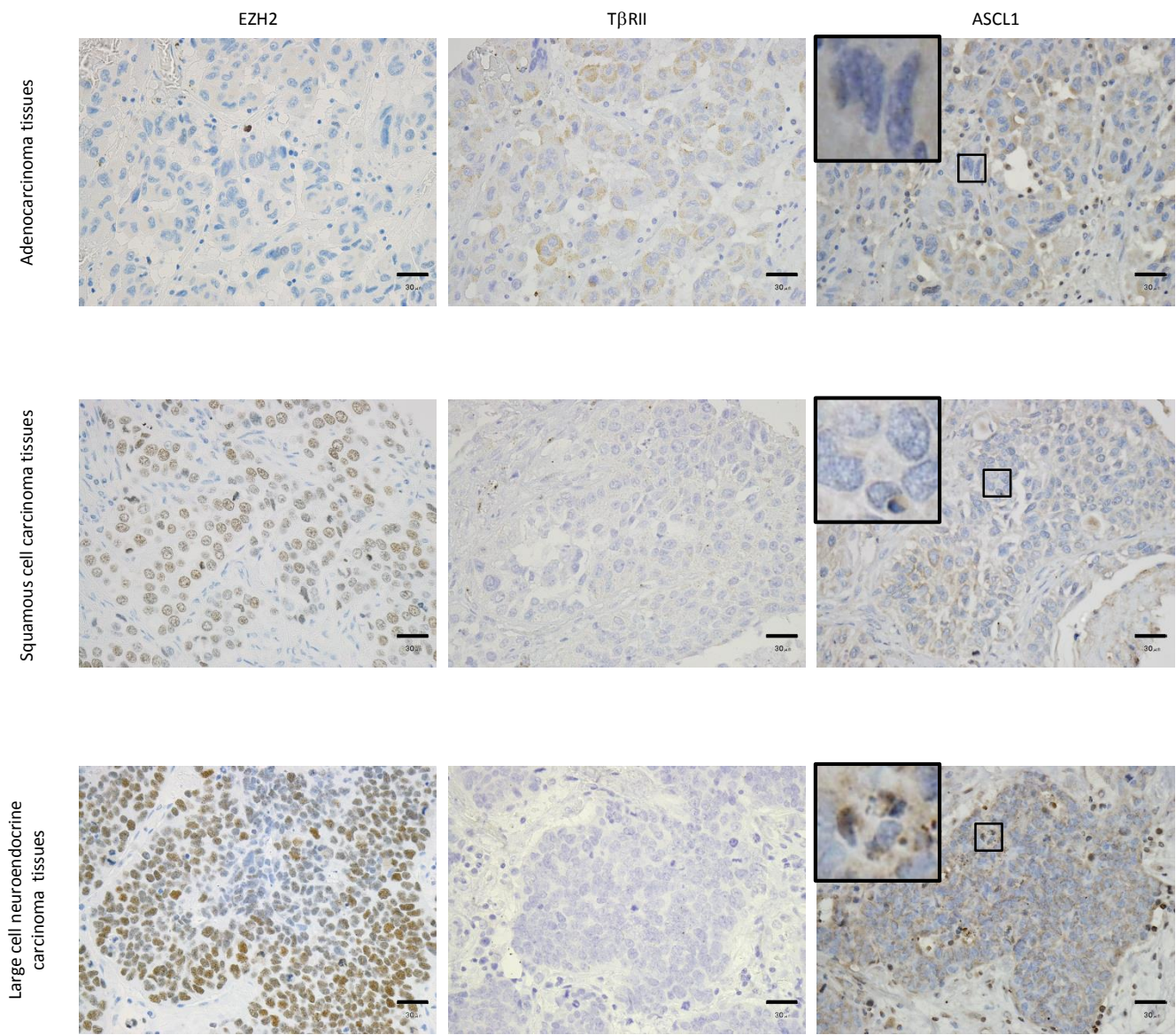

Supplement: Supplementary Figures [file celldisc201526-s1.pdf]
