# Supplementary material for: EZH2 promotes progression of small cell lung cancer by suppressing the TGF-β-Smad-ASCL1 pathway
Source: Cell Discov. 2015 Sep 22;1:15026–. doi: 10.1038/celldisc.2015.26 (PMC4860843; doi:10.1038/celldisc.2015.26)
Supplement: Supplementary Information [file celldisc201526-s2.pdf]

Supplementary Information

Supplementary Tables

Table S1. Immunohistochemical analysis samples in Figure 7.

Tissue	# of total samples	# of samples (individual specimens)	# of samples (TMA)
Normal	12	12	0
SCLC	19	13	6
Ad	44	0	44
Sq	41	0	41
LCNEC	6	0	6

Table S2. The number of samples for each score described in Figure 7.

Molecule	Tissue	# of analyzed samples	*s* = 4	3 ≤ s < 4	2 ≤ *s* < 3	1 ≤ *s* < 2	0 < *s* < 1	*s* = 0	n.e.
EZH2	Normal	12	0	0	0	7	0	5	0
	SCLC	19	13	6	0	0	0	0	0
	Ad	44	1	8	3	17	7	8	0
	Sq	41	12	19	7	3	0	0	0
	LCNEC	6	3	2	1	0	0	0	0
TβRII	Normal	12	12	0	0	0	0	0	0
	SCLC	19	0	0	0	4	1	14	0
	Ad	43	1	4	5	12	3	18	1
	Sq	38	0	3	2	12	6	15	3
	LCNEC	6	0	0	0	0	0	6	0
ASCL1	Normal	12	0	0	0	7	0	5	0
	SCLC	18	1	0	8	8	0	1	1
	Ad	40	0	0	0	3	3	34	4
	Sq	40	0	0	0	1	5	34	1
	LCNEC	5	0	0	2	2	0	1	1

Normal, normal lung

SCLC, small cell lung cancer (small cell carcinoma)

Ad, adenocarcinoma

Sq, squamous cell carcinoma

LCNEC, large cell neuroendocrine carcinoma.

n.e., not evaluated.

Table S3. Correlation coefficient between each two of EHZ2, TβRII and ASCL1 in all tissues in Figure 7.

	EZH2	TβRII	ASCL1
**EZH2**	1	-0.558 ***	0.279 *
**TβRII**	-0.558 ***	1	-0.091
**ASCL1**	0.279 **	-0.091	1

Pearson correlation coefficient test was performed.

**P < 0.01; ***P < 0.001.

Table S4. Oligonucleotide sequences for PCR experiments.

Experiment	Gene	Orientation	Sequences (5' to 3')
qRT-PCR	*ASCL1*	Forward	TCTTACGACCCGCTCAGCCCC
		Reverse	AGGTTGTGCGATCACCCTGCTT
	CDC25A	Forward	GCCTGTCACCAACCTGAC
		Reverse	CCAGGAGAATCTAGACAGAAACC
	CDKN1A	Forward	AGTGGACAGCGAGCAGCTGA
		Reverse	CGAAGTTCCATCGCTCACGG
	CDKN2B	Forward	CCGCCCACAACGACTTTATT
		Reverse	CAGCCTTCATCGAATTAGGTG
	EED	Forward	GTGACGAGAACAGCAATCCAG
		Reverse	TATCAGGGCGTTCAGTGTTTG
	EZH2	Forward	TCATGCAACACCCAACACTT
		Reverse	GCTCCCTCCAAATGCTGGTA
	GAPDH	Forward	GAAGGTGAAGGTCGGAGTC
		Reverse	GAAGATGGTGATGGGATTTC
	MYC	Forward	CCACACATCAGCACAACTACGC
		Reverse	CGGTTGTTGCTGATCTGTCTCA
	MYCL	Forward	GAGCGAGGGAGCGGACATGGA
		Reverse	GCGCCGTGGAGCGGTAGAAAT
	NCAM1	Forward	GTGCGTAGCCATGCCCGTGT
		Reverse	GCCCTGTAGCTTTGGGGCATATTG
	SMAD2	Forward	CCGACACACCGAGATCCTAAC
		Reverse	GAGGTGGCGTTTCTGGAATATAA
	SMAD3	Forward	TGGACGCAGGTTCTCCAAAC
		Reverse	CCGGCTCGCAGTAGGTAAC
	SMAD4	Forward	AAAACGGCCATCTTCAGCAC
		Reverse	AGGCCAGTAATGTCCGGGA
	SMAD7	Forward	CTGCAGACTGTCCAGATGCTGTG
		Reverse	GGCTCCAGAAGAAGTTGGGAATCTGA
	SUZ12	Forward	AGGCTGACCACGAGCTTTTC
		Reverse	GGTGCTATGAGATTCCGAGTTC
	SYP	Forward	GCCGCCAGACAGGGAACACA
		Reverse	CAGGAAGCCGAACACCACCGA
	TGFBR1	Forward	GCTGTATTGCAGACTTAGGACTG
		Reverse	TTTTTGTTCCCACTCTGTGGTT
	TGFBR2	Forward	GCTGTATGGAGAAAGAATGACGA
		Reverse	CATGAAGAAAGTCTCACCAGGC
ChIP-qRT-PCR	*ASCL1* (locus 1)	Forward	TTTTAACTTCCGTCAGGGCTCC
		Reverse	AGAACTTGGGTGCAGGAACA
	ASCL1 (locus 2)	Forward	TGTGGTCGCTTTGAGACACT
		Reverse	TTTACACGCAGCTCACGCTA
	HBB	Forward	GGGCTGAGGGTTTGAAGTCC
		Reverse	CATGGTGTCTGTTTGAGGTTGC
	HPRT1	Forward	TGTTTGGGCTATTTACTAGTTG
		Reverse	ATAAAATGACTTAAGCCCAGAG
	TGFBR2 (locus1)	Forward	CAGCTGAAAGTCGGCCAAAG
		Reverse	AGCCCCTAGCTCTCTCGTAG

ChIP-qRT-PCR	*TGFBR2* (locus2)	Forward	GCTCCCATGTTACCCCGTTT
		Reverse	TGGGAGTCACCTGAATGCTT
	TGFBR2 (locus3)	Forward	ATGGTGCCAAGAGAACGTGT
		Reverse	ACTCCTGGTCTTCGTGGATTG
	TGFBR2 (locus4)	Forward	CTCAATTTCACAGGAGACTGGAGA
		Reverse	TGGTGGTCTTTGCCATGATACA

Table S5. Oligonucleotide sequences for shRNA lentivirus vectors.

shRNA	Orientation	Sequences (5' to 3')
shNTC	Sense	GATCCCCGCGCGCTTTGTAGGATTCGACGTGTGCTGTCCGTCGAATCCTACAAAGCGCGCTTTTTGGAAAT
	Anti-sense	CTAGATTTCCAAAAAGCGCGCTTTGTAGGATTCGACGGACAGCACACGTCGAATCCTACAAAGCGCGCGGG
shASCL1 #1	Sense	GATCCCCGGAAAGCTCTGCCAAGATGACGTGTGCTGTCCGTCATCTTGGCAGAGCTTTCCTTTTTGGAAAT
	Anti-sense	CTAGATTTCCAAAAAGGAAAGCTCTGCCAAGATGACGGACAGCACACGTCATCTTGGCAGAGCTTTCCGGG
shASCL1 #2	Sense	GATCCCCGCTATTACCTCTGCATATTACGTGTGCTGTCCGTAATATGCAGAGGTAATAGCTTTTTGGAAAT
	Anti-sense	CTAGATTTCCAAAAAGCTATTACCTCTGCATATTACGGACAGCACACGTAATATGCAGAGGTAATAGCGGG
shASCL1 #3	Sense	GATCCCCGAGAGACATGGCTTTCAGAACGTGTGCTGTCCGTTCTGAAAGCCATGTCTCTCTTTTTGGAAAT
	Anti-sense	CTAGATTTCCAAAAAGAGAGACATGGCTTTCAGAACGGACAGCACACGTTCTGAAAGCCATGTCTCTCGGG
shEZH2 #1	Sense	GATCCCCGGGAGAGAACAATGATAAAACGTGTGCTGTCCGTTTTATCATTGTTCTCTCCCTTTTTGGAAAT
	Anti-sense	CTAGATTTCCAAAAAGGGAGAGAACAATGATAAAACGGACAGCACACGTTTTATCATTGTTCTCTCCCGGG
shEZH2 #2	Sense	GATCCCCGCACTTACTATGACAATTTACGTGTGCTGTCCGTAAATTGTCATAGTAAGTGCTTTTTGGAAAT
	Anti-sense	CTAGATTTCCAAAAAGCACTTACTATGACAATTTACGGACAGCACACGTAAATTGTCATAGTAAGTGCGGG
shSmad4	Sense	GATCCCCAAGCAATGGAACACCAATACTCAGGGTGTGCTGTCCCCTGAGTATTGGTGTTCCATTGCTTTTTTTGGAAAT
	Anti-sense	CTAGATTTCCAAAAAAAGCAATGGAACACCAATACTCAGGGGACAGCACACCCTGAGTATTGGTGTTCCATTGCTTGGG

Table S6. Oligonucleotide sequences for siRNA.

Gene	Sequence number	Sequences (5' to 3')
**NTC**	#1	UGGUUUACAUGUCGACUAA
	#2	UGGUUUACAUGUUUUCUGA
	#3	UGGUUUACAUGUUUUCCUA
	#4	UGGUUUACAUGUUGUGUGA
ASCL1	#1	CCAAUAAGCUGUAGACAUU
	#2	CCUGCAUCUUUAGUGCUUU
	#3	CCUUACAACUGCAAUUUUC
	#4	CUACCAUUUUCAUUAUAGA

Supplementary Methods

Cell culture

H82 cells and H441 cells were maintained in RPMI 1640 medium (Life Technologies) supplemented with 10% fetal bovine serum (FBS) (HyClone). H146 cells and H209 cells were maintained in RPMI 1640 medium supplemented with 10% FBS, 10 mM HEPES (Life Technologies), and 1 mM sodium pyruvate (Life Technologies). H345 cells were maintained in Dulbecco's Modified Eagle Medium (DMEM)/ Nutrient Mixture F-12 (1:1) medium (Life Technologies) supplemented with 5 μg/ml insulin (Sigma-Aldrich), 5 μg/ml transferrin (Sigma-Aldrich), and 30 nM sodium selenite (Sigma-Aldrich), 10 nM hydrocortisone (Sigma-Aldrich), 10 nM β-estradiol (Sigma-Aldrich), 15 mM HEPES, and 4.5 mM L-glutamine (Life Technologies). A549 cells and HaCaT cells were maintained in DMEM (Life Technologies) supplemented with 10% FBS.

Immunoblotting

The primary antibodies and dilutions were: anti-TβRII (Santa Cruz, sc-17792, 1:100), anti-EZH2 (Cell Signaling, 5246, 1:1000), anti-H3K27me3 (Monoclonal antibody, MABI0323, 1:1000), anti-H3S10p (Cell Signaling, 3377, 1:2000), anti-H3 (total) (Cell Signaling, 4499, 1:2000), anti-ASCL1 (Abcam, ab74065, 1:900), anti-pRB (BD Pharmingen, 554136, 1:1000) anti-phospho-Smad2 (Cell Signaling, 3108, 1:500), anti-PARP (Cell Signaling, 9542, 1:1000), and anti-α-tubulin (Sigma Aldrich, T6199, 1:10000). The secondary antibodies were: HRP-linked anti-mouse IgG (Cell Signaling, 7076, 1:10000) and HRP-linked anti-rabbit IgG (Cell Signaling, 7074, 1:10000). Each primary antibody was diluted in TBS-T buffer (50 mM Tris-HCl (pH 7.4), 150 mM NaCl, and 0.1% Tween-20) and incubated with protein-transferred membrane for 16 h at 4°C. Each secondary antibody was diluted in TBS-T buffer and incubated with the membrane for 1 h at room temperature.

ChIP

The anti-EZH2 antibody (5 μg), the anti-Smad2/3 antibody (5 μg) or the same amount of isotype control (IgG1 antibody, R & D Systems) was bound to either Dynabeads Protein A for immunoprecipitation (1.5 mg, Life Technologies) or Dynabeads coated with sheep anti-mouse IgG (4×10^7 beads, Life Technologies) in 0.5% BSA for 24 h at 4°C. Cells were fixed with 1% formaldehyde, suspended in sonication/elution buffer (50 mM Tris-HCl (pH 8.0), 10 mM EDTA (pH 8.0), 1% SDS, and

1 × cOmplete Protease Inhibitor Cocktail), and subjected to sonication by Bioruptor (Cosmobio, high intensity, 3 cycles of 30 sec of sonication and 30 sec of incubation). The sonicated DNA fragments were incubated with the antibody-Dynabeads complex in immunoprecipitation buffer (20 mM Tris-HCl (pH 8.0), 2 mM EDTA (pH 8.0), 1% Triton-X100, 150 mM NaCl, and 1 × cOmplete Protease Inhibitor Cocktail) for 24 h at 4°C. Then, immunoprecipitated samples were washed with ChIP RIPA wash buffer (50 mM HEPES-KOH (pH 7.0), 500 mM LiCl, 1 mM EDTA, 0.7% sodium deoxycholate, and 1% Nonidet P-40) 5 times, followed by wash with TE buffer (10 mM Tris-HCl (pH 8.0), and 1 mM EDTA (pH 8.0)) once, and the DNA fragments were eluted with sonication/elution buffer for 16 h at 65°C. The eluted DNA fragments were purified with the QIAquick PCR Purification Kit (Qiagen).

Immunohistochemistry

Primary antibodies were: anti-TβRII (Abcam, ab78419, 1:100), anti-EZH2 (Cell Signaling, 5246, 1:50), and anti-ASCL1 (Abcam, ab135273, 1:20). Each primary antibody was diluted in Blocking One (Nacalai Tesque) and incubated with sections for 16 h at 4°C, followed by incubation for 30 min at 40°C.

Immunofluorescence

Preparation of frozen sections was previously described (Kawabata et al., 2013). Briefly, tumor samples were frozen in acetone on dry-ice. The frozen sections were fixed with 4% paraformaldehyde, followed by permeabilization in 0.2% Triton-X100. Rat-anti-mouse CD31 antibody (BD Pharmingen, 550274, 1:400) and Alexa Fluor 488-conjugated anti-rat IgG antibody (Life Technologies, A-11006, 1:400) were used for immunostaining. Fluorescent images were captured with a BZ-9000 Fluorescence Microscope (Keyence). $CD31^+$ pixels were analyzed with Image J (National Institutes of Health).

Data analysis and filter criteria

The raw signal intensities of GSE32036 samples were normalized by quantile algorithm with 'preprocessCore' library package [S1] on Bioconductor software [S2]. We selected probes that call 'Detection $P < 0.05$' flag at least one sample. Then,

we applied Linear Models for Microarray Analysis (limma) package [S3] of Bioconductor software. The criteria of differentially expressed genes was that limma $P < 0.05$ and absolute log-fold-change ($|logFC|$) > 1. (Un-log-transformed intensities and ratio were shown on the table.) The heat map was generated by MeV software [S4]. We used a hierarchical clustering (HCL) method to sort the genes.

Supplementary References

S1. Bolstad BM, Irizarry RA, Astrand M, *et al.* A comparison of normalization methods for high density oligonucleotide array data based on variance and bias. *Bioinformatics* 2003; **19**:185-93.

S2. Gentleman RC, Carey VJ, Bates DM, *et al.* Bioconductor: open software development for computational biology and bioinformatics. *Genome Biol* 2004; **5**:R80.

S3. Smyth GK. Limma: linear models for microarray data. In: Gentleman R, Carey VJ, Dudoit S, eds. Bioinformatics and computational biology solutions using R and bioconductor. New York: Springer, 2005:397-420.

S4. Saeed AI, Sharov V, White J, *et al*. TM4: a free, open-source system for microarray data management and analysis. *BioTechniques* 2003; **34**:374-8.

Supplementary Figure Legends

Figure S1. Expression of *TGFBR2* is attenuated in SCLC.

Comprehensive gene expression analysis from the NCBI GEO database (GSE11969) shows expression of *TGFBR2* in normal lung tissues ($n = 5$) and SCLC tissues ($n = 9$). Data represent means. ***$P < 0.001$.

Figure S2. Inhibition of TGF-β signal promotes SCLC tumor formation.

(A) H146 cells were infected with lentivirus vectors encoding GFP (H146-GFP) or dnTβRII (H146-dnTβRII). GFP fluorescence was confirmed with fluorescence microscopy.

(B) Immunoblot of cell lysates probed with the indicated antibodies. H146-GFP cells and H146-dnTβRII cells were stimulated with TGF-β for 2 h.

(C) qRT-PCR analysis shows *SMAD7* expression of cells in (B). Data represent means ± SD. ***$P < 0.001$.

(D) Mice received subcutaneous transplants of H146-GFP cells ($n = 9$) or H146-dnTβRII cells ($n = 10$). Tumor volume was measured at the indicated time points. Data represent mean ± SEM. ***$P < 0.001$.

(E) Immunostaining of CD31 in tumor tissues dissected from mice in (D), which were sacrificed 6 weeks after subcutaneous transplantation. (Left panels) Representative images were captured for each mouse. Scale bars indicate 100 μm. (Right panel) Angiogenesis was quantified by counting the number of $CD31^+$ pixels. Data represent mean ± SD.

(F) Cell proliferation assay. H146 cells were stimulated with TGF-β for 6 days. Data represent mean ± SD. ***$P < 0.001$.

Figure S3. Expression of PRC2 components is up-regulated in SCLC.

Expression analyses of PRC2 components ~~in normal lung epithelial cells and lung cancer cells **(A)** and~~ in normal lung tissues and SCLC tissues, based on the data shown in Figure S1.~~A and S1B respectively.~~ Data represent means. **$P < 0.01$; ***$P < 0.001$.

Figure S4. SCLC cells and tissues express higher levels of ASCL1 than other neuroendocrine-related genes.

(A) Expression analysis of neuroendocrine-related genes in normal lung epithelial cells and lung cancer cells, based on the

data shown in Figure 1D. The color indicates the distance from the median of each row. **(B)** Expression analysis of neuroendocrine-related genes in normal lung tissues and SCLC tissues, based on the data shown in Figure S1. Data represent means. *$P < 0.05$; **$P < 0.01$; ***$P < 0.001$.

Figure S5. ASCL1 is directly and specifically regulated by TGF-β in SCLC cells.

(A) qRT-PCR analysis shows expression of neuroendocrine-related genes (*MYCL*, *NCAM1*, and *SYP*). H345-GFP cells and H345-TβRII cells were stimulated with TGF-β for the indicated times. Data represent means ± SD.

(B) qRT-PCR analysis shows expression of *SMAD7* and neuroendocrine-related genes (*ASCL1*, *MYCL*, *NCAM1*, and *SYP*). H146 cells were stimulated with TGF-β for the indicated times. Data represent means ± SD.

(C) qRT-PCR analysis shows *ASCL1* expression in SCLC cells (H345-TβRII cells and H146 cells) and NSCLC cells (A549 cells and H441 cells), after TGF-β stimulation for 4 h. Data represent means ± SD. ***$P < 0.001$; n.d., not determined.

Figure S6. Expression of EZH2, TβRII, and ASCL1 in various types of human lung cancer tissues.

Human lung cancer tissue samples (top: adenocarcinoma, middle: squamous cell carcinoma, and bottom: large cell neuroendocrine carcinoma) were stained with (left to right) anti-EZH2 antibody, anti-TβRII antibody, and anti-ASCL1 antibody. Representative images are shown. Insets show ASCL1 staining in the boxed region at high magnification. Scale bars indicate 30 μm.
